# Evolution of population structure in an estuarine‐dependent marine fish

**DOI:** 10.1002/ece3.4936

**Published:** 2019-02-26

**Authors:** Christopher M. Hollenbeck, David S. Portnoy, John R. Gold

**Affiliations:** ^1^ Marine Genomics Laboratory, Department of Life Sciences Texas A&M University ‐ Corpus Christi Corpus Christi Texas; ^2^ Present address: Scottish Oceans Institute University of St. Andrews St. Andrews, Fife UK

**Keywords:** historical biogeography, isolation by adaptation, population genomics, RADseq

## Abstract

Restriction site‐associated DNA (RAD) sequencing was used to characterize neutral and adaptive genetic variation among geographic samples of red drum, *Sciaenops ocellatus*, an estuarine‐dependent fish found in coastal waters along the southeastern coast of the United States (Atlantic) and the northern Gulf of Mexico (Gulf). Analyses of neutral and outlier loci revealed three genetically distinct regional clusters: one in the Atlantic and two in the northern Gulf. Divergence in neutral loci indicated gradual genetic change and followed a linear pattern of isolation by distance. Divergence in outlier loci was at least an order of magnitude greater than divergence in neutral loci, and divergence between the regions in the Gulf was twice that of divergence between other regions. Discordance in patterns of genetic divergence between outlier and neutral loci is consistent with the hypothesis that the former reflects adaptive responses to environmental factors that vary on regional scales, while the latter largely reflects drift processes. Differences in basic habitat, initiated by glacial retreat and perpetuated by contemporary oceanic and atmospheric forces interacting with the geomorphology of the northern Gulf, followed by selection, appear to have led to reduced gene flow among red drum across the northern Gulf, reinforcing differences accrued during isolation and resulting in continued divergence across the genome. This same dynamic also may pertain to other coastal or nearshore fishes (18 species in 14 families) where genetically or morphologically defined sister taxa occur in the three regions.

## 1 introduction

Genetic variation within and among contemporaneous populations is the result of the interplay among historical events and evolutionary‐genetic forces such as genetic drift, selection, and gene flow (migration) that change or maintain gene frequencies and act at the genomic, individual, and population levels (Garant, Forde, & Hendry, 2007). A key tenet of population genetics is that differences in selectively “neutral” loci arise from the balance between genetic drift and gene flow and can be used to study demographic and historical processes; alternatively, loci under the influence of selection often exhibit “outlier” patterns of variation that can be used to infer the role of selection operating in local and/or regional environments (Luikart, England, Tallmon, Jordan, & Taberlet, 2003). This separation of genetic variation into neutral and outlier components provides a more complete view of population structure and of processes that generate and maintain independent subpopulations or demes (Allendorf, Hohenlohe, & Luikart, 2010; Luikart et al., 2003). This is especially relevant to conservation and management of highly exploited species (Fraser & Bernatchez, 2001; Waples, 1995), in part to identify units for management and in part to identify genes or genomic regions putatively involved in locality‐specific adaptations that allow individuals to survive and reproduce successfully (Bradbury et al., 2013). Identifying adaptive variation also is important for (a) monitoring the potential of species to respond to environmental change and/or exploitation (Allendorf, England, Luikart, Ritchie, & Ryman, 2008; Schwartz, Luikart, & Waples, 2007) and (b) increasing power to discriminate among demes that simultaneously experience strong, localized directional selection and high gene flow or that have diverged too recently for genetic drift to have appreciably changed neutral allele frequencies (Nielsen, Hemmer‐Hansen, Larsen, & Bekkevold, 2009). The purpose of this study was to better understand how historical events and microevolutionary forces (genetic drift, gene flow, and selection) have interacted to generate geographic partitioning of genetic variation in red drum, *Sciaenops ocellatus*, a highly exploited estuarine‐dependent marine fish.

Briefly, red drum inhabit coastal waters of the northwestern Atlantic Ocean (Atlantic) and Gulf of Mexico (Gulf) and support large recreational fisheries in both regions (NOAA, 2017). Adults spawn in nearshore waters and larvae settle into bays and estuaries where they remain until maturity between the ages of three and six years. The species is iteroparous, with a reproductive life span of >40 years (SEDAR, 2015; Wilson & Nieland, 1994). The species is cultured in the United States for commercial sale (Treece, 2017) and for state‐managed stock‐enhancement programs (SCDNR, 2018; Tringali et al., 2008; Vega, Neill, Gold, & Ray, 2011).

Assessment of genetic structure of red drum has been ongoing for nearly 30 years and has included studies of allozymes (Bohlmeyer & Gold, 1991), mitochondrial DNA (Gold, Richardson, & Turner, 1999; Seyoum, Tringali, Bert, Tringali, Bert, McElroy, & Stokes, 2000), and microsatellites (Gold & Turner, 2002). The consensus among these studies is that weak but significant genetic divergence exists between red drum in the Atlantic and Gulf and that an isolation‐by‐distance pattern occurs among red drum in the Gulf. Gold, Burridge, and Turner (2001) hypothesized that population structure in the Gulf is best described by a modified, one‐dimensional, stepping‐stone model where gene exchange occurs primarily between adjacent bays or estuaries. This model is consistent with tag‐and‐recapture studies that have shown spawning site fidelity and natal homing (Lowerre‐Barbieri, Burnsed, & Bickford, 2016; Patterson, McBride, & Julien, 2004). However, bays and estuaries along the Atlantic and Gulf coasts exhibit heterogeneity in space and time in a wide variety of abiotic factors (e.g., temperature, salinity, oxygen level) and anthropogenic stressors (EPA, 1998, 1999). The environmental and ecological differences in U.S. waters inhabited by red drum, along with their life history and reproductive behavior suggest the potential for important localized and/or regional adaptive genetic differences could be large.

We used restriction site‐associated DNA (RAD) sequencing to characterize patterns of neutral and adaptive genetic variation within and among samples of red drum juveniles collected in estuarine environments spanning the species range in U.S. waters. We also used a linkage map developed for red drum (Hollenbeck et al., 2017) to identify chromosomal locations of outlier loci to assess chromosomal architecture of putatively adaptive variation.

## 2 materials and methods

### 2.1 Materials

**Figure 1:**
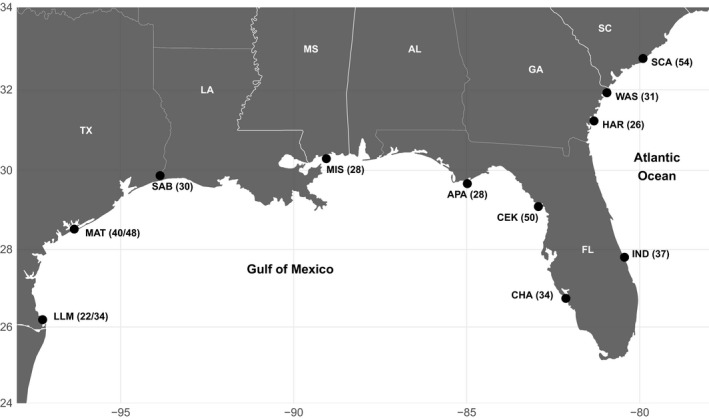
Map of the 11 localities where red drum were sampled. Numbers in parentheses represent the final filtered sample size for each locality. Sample sizes for LLM and MAT represent each of two temporal samples from each locality taken in the spring of 2008 (first value) and the winter of 2014/2015 (second value). Acronyms for states are as follows: TX (Texas), LA (Louisiana), MS (Mississippi), AL (Alabama), FL (Florida), GA (Georgia), and SC (South Carolina).

A total of 563 juvenile red drum were sampled between 2008 and 2015 from 11 estuarine localities in the Atlantic and Gulf (Figure 1; Supporting information Table S1). Sample localities included four in the Atlantic (Charleston Harbor, South Carolina, SCA; Wassaw Sound, WAS; Hampton River, HAR; and Indian River, IND) and seven in the Gulf (Charlotte Harbor, CHA; Cedar Key, CEK; Apalachicola, APA; Biloxi Bay, Mississippi, MIS; Sabine Lake, SAB; Matagorda Bay, MAT; and Lower Laguna Madre, LLM). Two localities in Texas, LLM and MAT, were sampled in different years to assess temporal stability of genomic variation.

Double‐digest RAD libraries were prepared as in Portnoy, Puritz, Hollenbeck et al. (2017) and sequenced on three lanes of an Illumina HiSeq 2000 DNA sequencer. Demultiplexing was performed with the program *process_radtags* from the *Stacks* package (Catchen, Amores, Hohenlohe, Cresko, & Postlethwait, 2011), and read mapping and SNP calling with the *dDocent* package (Puritz, Hollenbeck, & Gold, 2014), following Hollenbeck et al. (2017). SNP calls were filtered using VCFtools (Danecek et al., 2011). Genotypes of five individuals were duplicated in different RAD libraries and their genotypes compared to assess for systematic genotyping error. Suspected first‐generation hatchery‐produced individuals in samples from Texas waters, derived from the TPWD (Texas Parks and Wildlife) stock‐enhancement program (Vega, Neil, Gold & Ray, 2011), were identified genetically and discarded. Contiguous sequences alignments (contigs) were rigorously filtered and complex polymorphisms decomposed to individual SNPs or indels. The resulting dataset was phased into constituent haplotypes, using rad_haplotyper.pl (Willis, Hollenbeck, Puritz, Gold, & Portnoy, 2017), creating a dataset of multi‐allelic, SNP‐containing haplotypes with one diploid genotype per individual per locus (genepop file available at https://github.com/chollenbeck). Additional details of the above procedures are given in Supplementary Information.

### 2.2 Statistical analysis

Temporal stability of allele frequencies was assessed by comparing samples obtained from the same localities (MAT and LLM) in different years (LLM 2008, LLM 2014; MAT 2008, MAT 2015). Pairwise *F*_ST_ was calculated in arlequin (Excoffier & Lischer, 2010) and significance assessed using 10,000 permutations. Pairwise *F*_ST_ did not differ significantly between years at either location: LLM (*F*_ST_ = −0.00017, *p* = 0.767) and MAT (*F*_ST_ = 0.00048, *p* = 0.179).

Principal components analysis (PCA), as implemented in adegenet (Jombart, 2008) in R (R Core Team, 2016), was used to assess patterns of variation among individuals across all loci. The PCA revealed three distinct clusters (Supporting information Figure S1), corresponding to samples from the northwestern Gulf (LLM, MAT, SAB, MIS), northeastern Gulf (APA, CEK, and CHA), and Atlantic (IND, HAR, WAS, and SCA). The PCA output confirmed the similarity of the temporal samples from LLM and MAT; the 2008 samples were removed from subsequent analyses.

To account for false positives in outlier detection, the presence of loci putatively under selection was assessed by using three *F*_ST_ outlier‐detection methods as recommended by Hoban et al. (2016): the *fdist* method (Beaumont & Nichols, 1996) implemented in lositan (Antao, Lopes, Lopes, Beja‐Pereira, & Luikart, 2008 ); a modification of the *fdist* method implemented in arlequin; and bayescan (Foll & Gaggiotti, 2008 ). For these analyses, comparisons were made among geographic samples. Loci with a global, major‐allele frequency above 0.95 were excluded from all outlier‐detection approaches because low minor allele frequencies can bias results (Roesti, Salzburger, & Berner, 2012). Additional details are given in Supplementary Information.

The dataset was subdivided into “outlier” and “neutral” components. The outlier dataset consisted of all loci identified as outliers under directional selection by at least one of the approaches; the neutral dataset consisted of remaining loci. All loci detected as outliers due to balancing selection had negative *F*_ST_ values, suggesting that mean *F*_ST_ in the dataset was too low for reliable detection of balancing selection (Narum & Hess, 2011); consequently, outlier loci identified as under balancing selection were treated as neutral loci.

**Figure 2:**
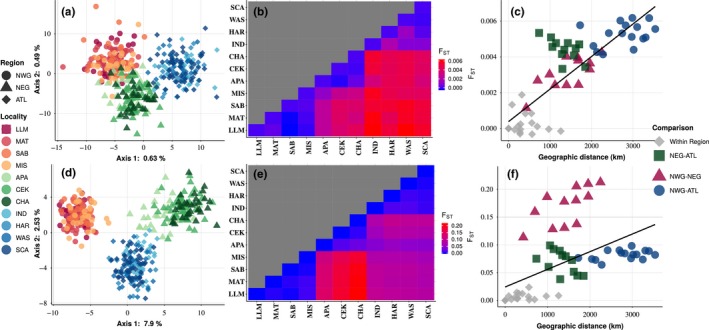
Population structure of red drum, evaluated with datasets consisting of only neutral loci (*n* = 1,393; panels a‐c) and only outlier loci (*n* = 146; panels d‐f). Column 1 (panels a, d) displays principal components analysis (PCA), using each dataset. Column 2 (panels b, e,) shows a heat map of pairwise *F*_ST_ estimates for each locality, using each dataset; blue squares represent low *F*_ST_ values, and red squares represent high *F*_ST_ values between localities. Localities (left to right) are organized geographically (west to east) along the coastline. Column 3 (panels c, f) shows the relationship between genetic distance (*F*_ST_; *y*‐axis) and geographic distance (km; *x*‐axis) for each pair of localities, using each dataset. Pairings between localities in different regions are highlighted by different colors (within region comparisons = gray; northeastern Gulf vs. Atlantic = green; northwestern Gulf vs. northeastern Gulf = red; northwestern Gulf vs. Atlantic = blue). The black line in each panel shows the best linear fit for the relationship.

Neutral and outlier datasets were separated and PCA implemented on each. The same three regional groupings (northwestern Gulf—NWG; northeastern Gulf—NEG; and Atlantic—ATL) were evident in PCA output of both datasets (Figure 2a, d). Homogeneity of the three regional groupings and of localities within each region was tested using hierarchical analysis of molecular variance (amova) implemented arlequin; significance of variance components was assessed using 10,000 permutations. Estimates of pairwise *F*_ST_ between localities and between regions (all individuals within regions pooled) were generated using arlequin, with significance assessed as above. Existence of an isolation‐by‐distance effect was assessed using Mantel tests as implemented in the *vegan* package (Oksanen et al., 2015) in R, using a matrix of pairwise *F*_ST_ values (Supporting information Table [Link], [Link]) coded as *F*_ST_/(1 − *F*_ST_), and a pairwise matrix of approximate coastline, linear geographic distance (Supporting information Table [Link], [Link]). Gene diversity (*H*_e_) for each locus and locality was calculated using the R package *hierfstat* (Goudet, 2005); differences among regions (all individuals within regions pooled) were assessed in R, using a Friedman rank sum test.

### 2.3 Redundancy analysis

Redundancy analysis (RDA), modified for genetic data, following Meirmans (2015), was conducted in R on the neutral and outlier datasets separately to assess the influence of geographic distance and 49 environmental variables (Supporting information Table [Link], [Link]) on observed patterns of genetic variation. Significance of components of genetic variance explained by geography, environment, and the interaction between the two was tested using 1,000 permutations. Additional details of this approach are given in Supplementary Information.

### 2.4 High‐confidence outliers and GO‐analysis

A subset of neutral and outlier loci had been mapped previously onto individual red drum chromosomes (Hollenbeck et al., 2017). For each of these mapped loci, global *F*_ST _and pairwise *F*_ST_ between regions were estimated after Weir and Cockerham (1984), using the *pegas* package (Paradis, 2010) in R. A set of 67 high‐confidence outliers were identified by selecting loci that met at least one of the following criteria: The locus (a) was identified by all three outlier‐detection approaches; (b) was within two cM of another outlier locus; and (c) had a global *F*_ST_ of at least 0.1. RAD reference sequences for high‐confidence outlier loci were compared to the annotated draft genome of another sciaenid (the large yellow croaker, *Larimichthys crocea*, GenBank assembly L_crocea_1.0) and gene‐ontology (GO) term enrichment analysis performed to develop a list of candidate genes potentially under the influence of selection.

## 3 results

A summary of data filtering is presented in Supporting information Table [Link], [Link]. The final dataset consisted of genotypes for 462 individuals at 1,539 (haplotyped) loci, consisting of 2,860 SNPs. The three outlier‐detection methods identified a total of 143 outliers (Supporting information Figure [Link], [Link]) putatively under directional selection (9.29% of all loci). lositan was the least conservative (143 loci), bayescan was intermediate (85 loci), and arlequin was the most conservative (52 loci). All 52 outliers identified by arlequin also were identified by the other two methods. The final outlier and neutral datasets consisted of 143 and 1,396 loci, respectively.

### 3.1 Statistical analysis

For neutral loci, the two largest principal components explained 0.63 and 0.49 percent of the total genetic variance, respectively (Figure 2a). Hierarchical amova (Table 1), based on neutral loci, revealed significant divergence among regions (*F*_CT_ = 0.004, *p* = 0.0003), but not among localities within regions (*F*_SC_ = 0.0002, *p* = 0.86). Except for MIS (NWG) versus APA (NEG), pairwise *F*_ST_ estimates between localities in different regions differed significantly from zero (Supporting information Table [Link], [Link]). The relative magnitude of pairwise *F*_ST_ values between localities also was consistent with three geographic clusters (Figure 2b). *F*_ST_ estimates for all pairwise comparisons between localities within regions, except for IND versus HAR (*F*_ST_ = 0.002, *p* = 0.016), were nonsignificant. Estimates of pairwise *F*_ST_ between regional groupings (all individuals within regions pooled) revealed a linear geographic pattern; NWG versus ATL was the most divergent (*F*_ST_ = 0.005, *p* < 0.001), followed by NEG versus ATL (*F*_ST_ = 0.004, *p* < 0.0001), and NWG versus NEG (*F*_ST_ = 0.003, *p* < 0.0001). A Mantel test, based on neutral loci (Figure 2c), revealed a strong, significant relationship between geographic and genetic distance (*r* = 0.626, *p* = 0.001). Mean *H*_e_, based on neutral loci, did not differ significantly among regions (NWG = 0.395, NEG = 0.396, ATL = 0.396; *p* = 0.558).

**Table 1:** Hierarchical analysis of molecular variance (AMOVA), based on datasets containing only neutral loci (*n* = 1,396), and only outlier loci (*n* = 143). *df* = degrees of freedom; *F* = fixation index; *p* = probability that *F* = 0. Significant values (*p* < 0.05) are in bold; significance was assessed using 10,000 permutations

Level	df	Sum of Squares	Variance Component	% Variation	F	p
Neutral Loci						
Among regions	2	1,161.113	1.14519	0.42	**0.00419**	0.0003
Among localities within regions	8	2,214.294	0.06410	0.02	0.00024	0.8624
Within localities	789	214,799.223	272.24236	99.56	**0.00442**	<0.0001
Outlier Loci						
Among regions	2	1,688.779	3.02590	10.01	**0.10009**	0.0001
Among localities within regions	8	332.087	0.20466	0.68	**0.00752**	<0.0001
Within localities	789	21,303.392	27.00050	89.31	**0.10686**	<0.0001

**Figure 3:**
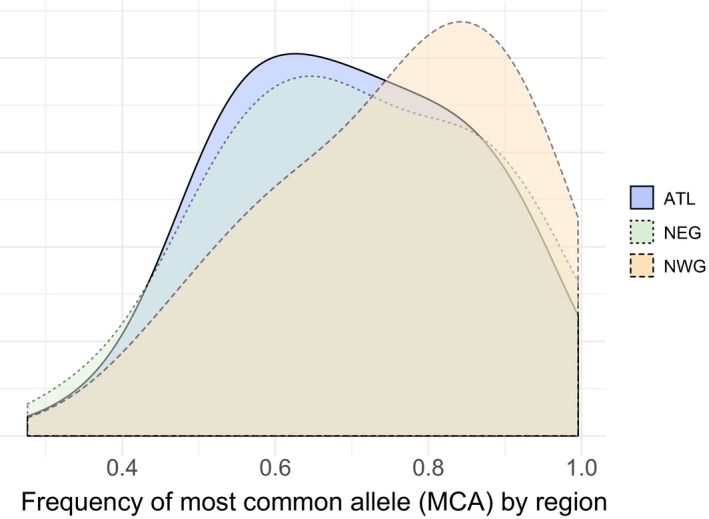
Density distribution (frequency) of the most common allele (MCA) at 67 high‐confidence, outlier loci, plotted by region

For outlier loci, the two largest principal components explained 7.9 and 2.5 percent of the total genetic variation, respectively (Figure 2d). Separation among the three clusters was more distinct than observed in the PCA of neutral loci (Figure 2a), with NWG especially distinct from NEG along Axis 1. Hierarchical amova (Table 1), based on outlier loci, revealed significant divergence among regions (*F*_CT_ = 0.100, *p* < 0.0001) and among localities within regions (*F*_SC_ = 0.0075, *p* < 0.0001). All pairwise *F*_ST_ estimates between localities in different regions and 12 of 15 pairwise *F*_ST_ estimates between localities within regions differed significantly from zero (Supporting information Table [Link], [Link]). Pairwise *F*_ST_ estimates between localities in different regions, however, were discordant with geography; estimates of *F*_ST_ between localities in NWG and in NEG were larger than estimates between localities in NWG and ATL, and overall divergence between NWG and NEG (*F*_ST_ = 0.168, *p* < 0.001) was over twice that of NWG versus ATL (*F*_ST_ = 0.076, *p* < 0.001) and NEG versus ATL (*F*_ST_ = 0.063, *p* < 0.001). Pairwise estimates of *F*_ST_ between regions using each of the 67 high‐confidence outliers revealed that 44 (66%) were highest between NWG and NEG, 12 (18%) between NWG and ATL, and 11 (16%) between NEG and ATL. To visualize differences among regions in allele frequencies of high‐confidence outliers, the frequency of the most common allele (MCA) for each of the 67 outliers was plotted for each region (Figure 3). MCAs overall had higher frequencies, including fixed differences, in the NWG.

A Mantel test, based on outlier loci, revealed a weak, but significant relationship between genetic and geographic distance (*r* = 0.127, *p* = 0.033). Inspection of the trend line, however, revealed a poor fit (Figure 2f). A p*ost hoc* Pearson's correlation (*r*) was used to test for correlations between genetic and geographic distance within localities and between localities in different regions; significance of *r* (*H*_0_: *r* = 0) was assessed using a Student's *t*, implemented in R. A significant positive correlation (*r* = 0.679, *p* = 0.015) was found only for the comparison between NWG and NEG. Mean *H*_e_, based on outlier loci, differed significantly among regions (NWG = 0.347, NEG = 0.396, ATL = 0.409; *p* = <0.001); pairwise Wilcoxon tests revealed that NWG differed significantly from NEG and ATL (*p* = 0.020 and 0.003, respectively), while NEG and ATL did not differ from one another (*p* = 0.643).

### 3.2 Redundancy analysis

**Figure 4:**
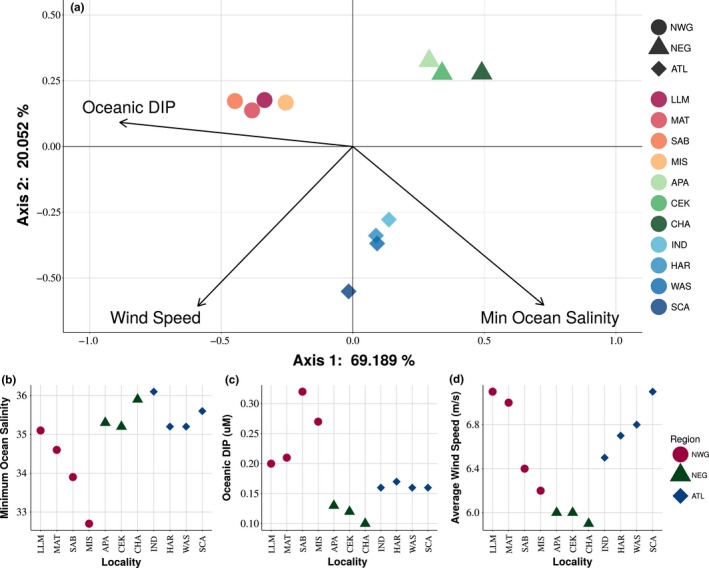
A biplot from redundancy analysis (RDA), using only outlier loci. Colored points represent individual localities; positions are indicative of the genetic relationships among localities, generated using correspondence analysis. Arrows represent the three environmental variables used in the RDA model; their directionality indicates the vector upon which each variable is correlated with genetic relationships among localities. Oceanic DIP is annual mean oceanic (outside of estuary) concentration of dissolved inorganic phosphates; Wind Speed is average estuarine wind speed; and Minimum Ocean Salinity is annual minimum oceanic (outside of estuary) salinity. Localities/regions are color coded

Forward selection of geographic distance variables for RDA resulted in selection of first‐ and second‐order polynomials in the model. Forward selection of environmental variables resulted in selection of three variables: mean oceanic (outside the estuary) concentration of dissolved inorganic phosphates (oceanic DIP), minimum oceanic salinity, and average wind speed. For neutral loci, the model explained a significant component of among‐locality genetic variance (*R* ^2^_Adj_ = 0.126, *p* = 0.007), but neither geography nor environmental variables, alone or together, contributed significantly (Table 2). For outlier loci, the model explained a large and significant component of among‐locality genetic variance (*R* ^2^_Adj_ = 0.695, *p* = 0.009); the component attributable to geography did not contribute significantly (*R* ^2^_Adj_  = 0.042, *p* = 0.079), whereas the component attributable to environmental variables did (*R* ^2^_Adj_ = 0.220, *p* = 0.015; Table 2). Inspection of RDA eigenvalues computed using outlier loci revealed that the largest RDA axes accounted for 69.19 and 20.05 percent of the genetic variance. An RDA biplot, based on outlier loci (Figure 4a), revealed that minimum oceanic salinity was lowest in the NWG and highest in the ATL, oceanic DIP was highest in the NWG and lowest in the NEG, and average wind speed was highest in the ATL and lowest in the NEG. Differences within regions (Figure 4b‐d) included lower minimum oceanic salinity, higher oceanic DIP, and reduced average wind speed from west to east in the NWG and higher wind speed from south to north in the ATL.

**Table 2:** Redundancy analysis (RDA), based on datasets containing only neutral loci (*n* = 1,396) or outlier loci (*n* = 143). Total Adjusted *R* ^2^ is the proportion of among‐locality genetic variance explained by independent variables (geography + environment + shared). Geography is the proportion of among‐locality genetic variance explained by geography; Environment is the proportion of among‐locality genetic variation explained by environmental variables; Shared is the proportion of among‐locality genetic variance explained by geography and environmental variables; and Residual is the proportion of among‐locality genetic variation not explained by the model. Significant values (*p* < 0.05) are in bold

	Neutral	Outlier
Total Adjusted R^2^	**0.126**	**0.695**
Geography	0.016	0.042
Environment	0.018	**0.220**
Shared	0.093	0.432
Residual	0.873	0.305

### 3.3 High‐confidence outliers and GO‐analysis

A total of 746 of 1,539 RAD loci (48.5%) were assigned a position on the red drum linkage map. Of the 67 high‐confidence outliers, 45 had been mapped, and of those, 39 were located within two cM of another high‐confidence outlier and organized into 15 clusters situated on ten different linkage groups (Supporting information Table [Link], [Link]).

Sixty‐two high‐confidence outliers had a significant hit to forty‐seven scaffolds in the genome of *L. crocea*. A total of 48 candidate genes, consisting of the gene nearest to the RAD locus with the highest *F*_ST_ on each scaffold, were selected. GO‐term enrichment analysis identified 27 biological processes. GO terms that were overrepresented significantly related primarily to neuron organization, development, and regulation. Broadening the candidate gene search to include all genes on the 47 *L. crocea* scaffolds within 100 kb of an outlier resulted in identification of an additional 215 candidate genes. Of these, 49 (22.8%) were associated with significant GO terms from the initial analysis. A complete list of significant GO terms is in Supporting information Table S7, and a complete summary of candidate genes is in Supporting information Table [Link], [Link].

## 4 discussion

Analyses of neutral and outlier SNP‐containing loci revealed three genetically distinct, regional clusters of red drum: one in the Atlantic (ATL) and two in the Gulf (NWG, NEG). Including red drum, genetically distinct populations in these three regions are now documented for 13 species of coastal or nearshore fishes (Supporting information Table [Link], [Link]), with separation occurring around the Florida Peninsula (the Florida vicariant zone; Neigel, 2009) and the northeastern Gulf (the Gulf suture‐zone; Portnoy & Gold, 2012). Concordance of genetic divergence among diverse taxa across geographic space likely reflects common separation by major geological or climatic events (Frankham, Ballou, & Briscoe, 2002). For coastal/nearshore marine species in the western North Atlantic, the most recent, major geological/climatic event was the last glacial retreat when suitable habitats (large river deltas, estuaries, warmer waters) became available ca. 6,000–10,000 years ago (Donoghue, 2011). Possible mechanisms that might have restricted gene flow among the three regions during and following the last glaciation are discussed elsewhere (Neigel, 2009; Portnoy & Gold, 2012; Portnoy et al., 2015; Seyoum et al., 2017, 2018).

Patterns and degree of genetic divergence in the two types of loci differed among the regions. Divergence in neutral loci indicated gradual genetic change across regions with divergence among regions following a linear pattern of isolation by distance. While the pattern of isolation by distance in the Gulf was detected in previous studies that employed fewer genetic markers (Gold et al., 1999; Gold & Turner, 2002), the significant genetic difference between NWG and NEG is a novel observation for red drum, demonstrating the increased power of next‐generation genotyping to resolve fine‐scale genetic differences (Luikart et al., 2003). Exchange of neutral loci between regions appears to be limited as tests of homogeneity were consistent with no exchange of neutral loci between the Atlantic and Gulf and limited exchange at the boundary between the two regional groups in the Gulf (between MIS and APA). Tests of homogeneity of neutral markers among localities within regions, with one exception, were nonsignificant, indicating either ongoing gene flow among localities or insufficient time for differences to accumulate. The exception was significant heterogeneity in neutral loci between IND and HAR (both in the Atlantic). Gold and Richardson (1994) also found genetic differences (in allozymes and mtDNA) between red drum from Mosquito Lagoon (part of the Indian River system) and red drum sampled off the Carolina coast. This suggests that genetically divergent populations of red drum may exist within regions.

Divergence of outlier loci differed from that of neutral loci in the proportion of genetic variation attributable to among‐region divergence (*F*_CT_ = 0.100 and *F*_CT_ = 0.004, respectively) and in significant heterogeneity among localities within regions (*F*_SC_ = 0.0075, *p* < 0.0001 and *F*_SC_ = 0.0002, *p* = 0.86, respectively). Overall, divergence in outlier loci was at least an order of magnitude greater than that in neutral loci, and divergence in outlier loci between NWG and NEG was at least twice that of divergence in outlier loci between NWG and ATL and NEG and ATL. The discordance in patterns of genetic divergence between outlier and neutral loci is consistent with the hypothesis that outlier loci reflect adaptive responses to environmental factors that vary on regional scales, while neutral loci reflect drift processes.

Hypotheses to explain genetic divergence between populations must account for mechanisms that initially reduced gene flow and those that maintain differences under present conditions (Neigel, 2009). All pairwise tests of homogeneity (neutral and outlier loci) between red drum in the Atlantic and Gulf were significant, consistent with a contemporaneous barrier to gene flow. One possible barrier may be the absence of suitable nearshore habitat along the southeast coast of the Florida peninsula (Gold & Richardson, 1998; Portnoy et al., 2014; Seyoum et al., 2018). Selection also may be involved as genetic divergence between red drum in the NEG and ATL in outlier loci (*F*_ST_ = 0.063) was an order magnitude greater than genetic divergence in neutral loci (*F*_ST_ = 0.004), and pairwise comparisons of *F*_ST_ between regions, based on outlier loci, identified 11 of 67 high‐confidence outliers as having the greatest impact on divergence between NEG and ATL. The RDA biplot and differences among regions in explanatory environmental variables suggest that wind speed may play a role in maintaining adaptive genetic differences between NEG and ATL.

Significant genetic divergence in neutral and outlier loci between red drum in NWG and NEG is consistent with a reduction in gene flow between the two regions. The homogeneity in allele distributions at neutral loci (*F*_ST_ = 0.001; *p* = 0.098) between MIS (NWG) and APA (NEG) suggests that gene flow occurs between these two localities; allele distributions at outlier loci, however, differed significantly between the two (*F*_ST_ = 0.114; *p* = 0), suggesting that gene flow between the two regions may be limited or limited to neutral loci. The degree of genetic divergence in outlier loci between the NEG and NWG suggests strong divergent selection stemming from adaptation to large, regional differences in habitat. The higher frequency and fixation of MCAs at outlier loci and significantly reduced genetic diversity (*H*_e_) suggest that selection may be more intense in the NWG. In addition, 12 of 15 possible pairwise comparisons, based on outlier loci, between localities within regions differed significantly. This and the significant positive correlation between divergence in outlier loci and geographic distance in the northern Gulf suggest that gene flow within regions may be impacted by localized selection driven by environmental/ecological differences operating at the scale of individual estuaries. Heterogeneity in genomic divergence between neutral and outlier loci in other organisms is documented and often hypothesized to indicate divergence with ongoing gene flow (Nosil, Funk, & Ortiz‐Barrientos, 2009). A cautionary note (Nosil, 2012), however, is that little to no divergence in neutral loci may simply reflect recent coancestry among populations and insufficient time for significant divergence in neutral loci to arise.

The nonrandom clustering of outlier loci on different chromosomes indicates selection operating at the genomic level. Such clusters of outlier loci (genomic islands of divergence; Nosil & Feder, 2012) occur when a small proportion of the genome diverges due to local adaptation and the genes involved become closely linked, reducing the rate at which genetic recombination breaks up locally adaptive genotype combinations (Yeaman, 2013). The phenomenon has been described in several species, including fishes (Hemmer‐Hansen et al., 2013; Larson et al., 2017), and has been associated with incipient speciation (Nosil & Feder, 2012). In the clusters of linked and unlinked outliers where RAD loci could be mapped to the *L. crocea* genome, overrepresented GO terms identified gene sets primarily involving neuron organization, development, and regulation. Clustering of these GO terms is consistent with the general observation that gene sets which share related functions tend to be concentrated within chromosomes (Dávila López, Martínez‐Guerra, & Samuelsson, 2010; Oliver & Misteli, 2005).

Despite strong evidence for natural selection at the genetic level, it is difficult to hypothesize about mechanisms of adaptation at the level of phenotype without further data. However, the potential role of nervous system development suggests that adaptive differences may be behavioral in nature or possibly the result of sensory (olfactory or visual) advantages in one region over another. In support of this, catenins, cadherins, and ptprz1 are thought to be important for learning and memory in vertebrates (Schrick et al., 2007; Takahashi et al., 2011) and have been shown to be important in olfactory and/or visual development (Akins & Greer, 2006; Babb et al., 2005; Osterhout, Stafford, Nguyen, Yoshihara, & Huberman, 2015; Yamagata, Weiner, & Sanes, 2002). It seems highly likely that some aspects of regional environments are promoting adaptive differences among red drum, possibly at early stages of development. The set of candidate genes identified in this study will be an excellent starting point for generating hypotheses and further targeted research into the operative mechanisms of selection.

The RDA biplot and the differences between the NWG and NEG in explanatory environmental variables indicate that higher levels of oceanic DIP and, to a lesser extent, lower levels of salinity differentiate red drum habitat in the NWG from that in the NEG. Accumulation of inorganic phosphate in coastal habitats stems from inflow of freshwater containing dissolved phosphate and inert phosphorus derived from weathering of continental rock (Frolich, 1988) and, more recently, from human effluents (Harrison, Bouwman, Mayorga, & Seitzinger, 2010). Freshwater inflow into the northern Gulf following glacial retreat (Wickert, 2016) and today is dominated by the Mississippi River and notable discharges occurring to the east and west of the Mississippi Delta (Supplemental Table 10). Increased riverine inflow also increases deposition of terrigenous sediments (Uncles & Mitchell, 2017), consistent with significant differences in continental shelf surface sediments between the region to the east of the Apalachicola River and south along the West Florida Shelf (mostly pre‐Quaternary carbonate‐rich layers) and to the west where surface sediments initially are a mix of sand and mud, with the area to the west of the Mississippi Delta containing a layer of mud roughly 8 m thick (Davis, 2017).

The basic differences in habitat between the NWG and NEG appear to be perpetuated in part by freshwater export pathways in the northern Gulf. Morey, Martin, O'Brien, Wallcraft, and Zavala‐Hildago (2003) identified seasonal pathways of freshwater discharged from the major rivers, primarily the Mississippi and the Atchafalaya, in the northern Gulf. Briefly, during the spring and summer, coastal winds move freshwater discharge to the east toward DeSoto Canyon, an old, deep, steep‐sided valley off the Florida Panhandle, that cuts through the continental shelf to the southwest and whose northeastern wall extends to ~25 m from shore (Davis, 2017; Harbison, 1968). Once in the canyon, the discharge water interacts with open‐ocean, mesoscale eddies intruding into the canyon from the Loop Current and is transported offshore (Morey et al., 2003). To the east, the steeply sloped (40°) Florida Escarpment separates the canyon from the carbonate‐dominated West Florida Shelf (Christeson et al., 2014), while to the west of the canyon terrigenous sediments become dominant (Hine, Dunn, & Locker, 2013). During the fall and winter, the winds shift to northeasterly, consistent with a westward/southward coastal current that transports the river discharge through the NWG and into waters off the coast of Mexico (Morey et al., 2003).

Based on the above, present‐day population structure of red drum in the northern Gulf appears, in part, to be a function of selection acting on individuals in subpopulations or demes that became separated following the last glaciation. Basic habitat differences, initiated by glacial retreat and perpetuated by contemporary oceanic and atmospheric forces interacting with the geomorphology of the northeastern Gulf basin, followed by selection, appear to have led to reduced gene flow across the region, reinforcing accrued differences and resulting in continued genomewide divergence. This process, termed isolation by adaptation or IBA (Nosil, Egan, & Funk, 2008), has been observed in animal and plant species (Van Bocxlaer, 2017; Funk, Egan, & Nosil, 2011; Mallet, Martos, Blambert, Martos, Blambert, Pailler, & Humeau, 2014) and, potentially, is a harbinger of incipient speciation (Schemske, 2010). The same dynamic may pertain to other coastal or nearshore fishes (18 species in 14 families) where genetically or morphologically defined sister taxa (species, subspecies, populations) occur in the three regions (Supplemental Table 9; Portnoy & Gold, 2012).

Hypotheses previously suggested to account for observed faunal/genetic discontinuities in the northeastern Gulf have involved either physical (e.g., a peninsula) or ecological (e.g., large freshwater inflow) barriers that restricted dispersal or secondary contact between different refugial populations following glacial retreat (Portnoy & Gold, 2012). Results of our study are consistent with the influence of physical (oceanic, atmospheric, basin geography) forces separating red drum in the northern Gulf but also demonstrate that selection has played a major role in reinforcing initial divergence. This role of selection can be assessed further in marine species when genetic and other discontinuities are observed in seemingly open systems where there are no apparent physical barriers to gene flow.

## conflicts of interest

None declared.

## author contributions

C.M.H. executed laboratory work, data analyses, and design of the study, and drafted the manuscript; D. S. P. executed data analysis and design of the study, and drafted the manuscript; J. R. G. procured the necessary funds, participated in fieldwork, data analyses, and design of the study, and drafted the manuscript.

## Data Availability

Additional data are provided in the electronic supplementary material and are also available at http://github.com/chollenbeck. Raw sequence reads are available under NCBI BioProject accession number PRJNA515285.
