# Supplementary figures and images for: Evolution of population structure in an estuarine‐dependent marine fish
Source: Ecol Evol. 2019 Feb 26;9(6):3141–52. doi: 10.1002/ece3.4936 (PMC6434539; doi:10.1002/ece3.4936)

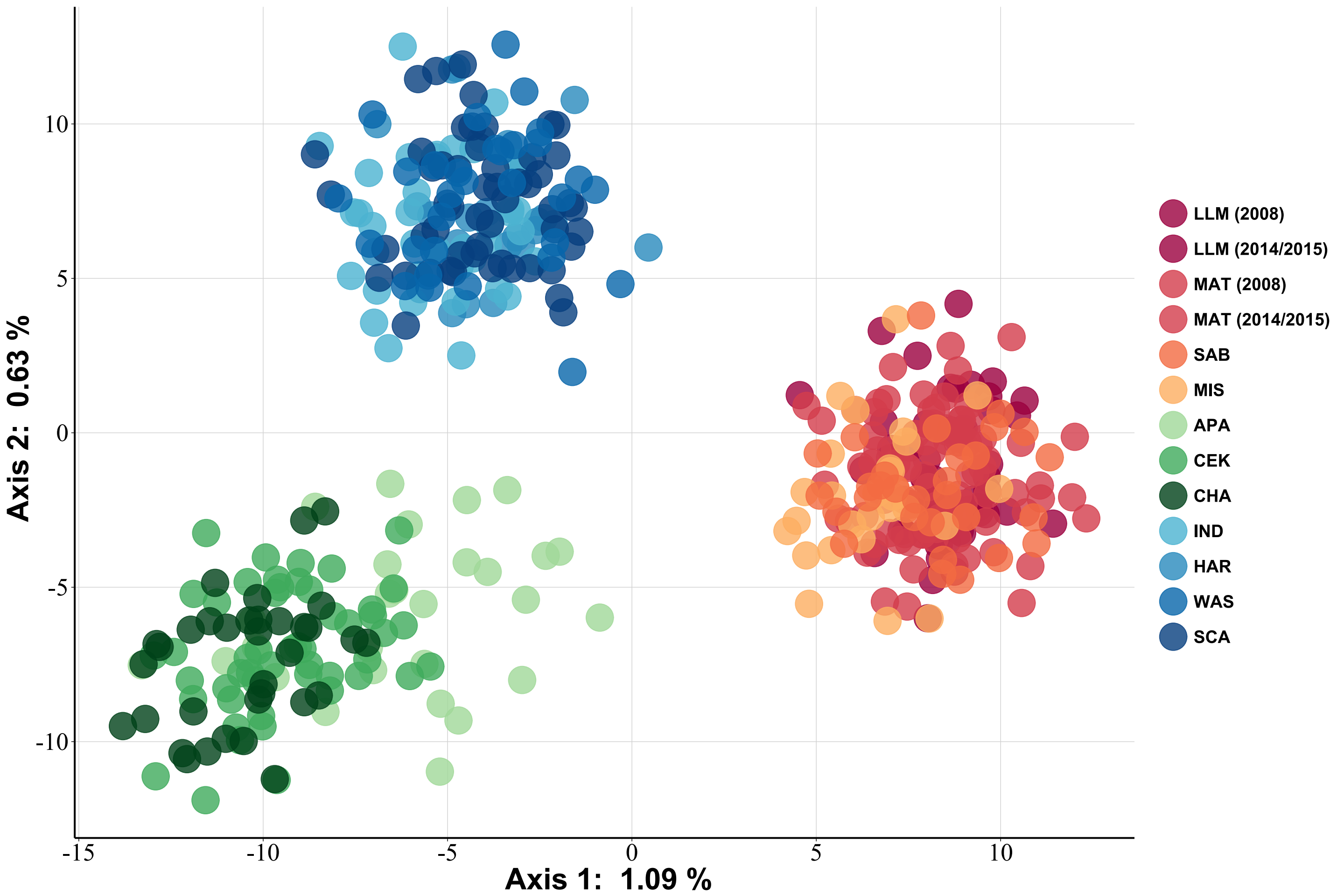

Supplement: Supplementary file 1 [file ECE3-9-3141-s001.pdf]

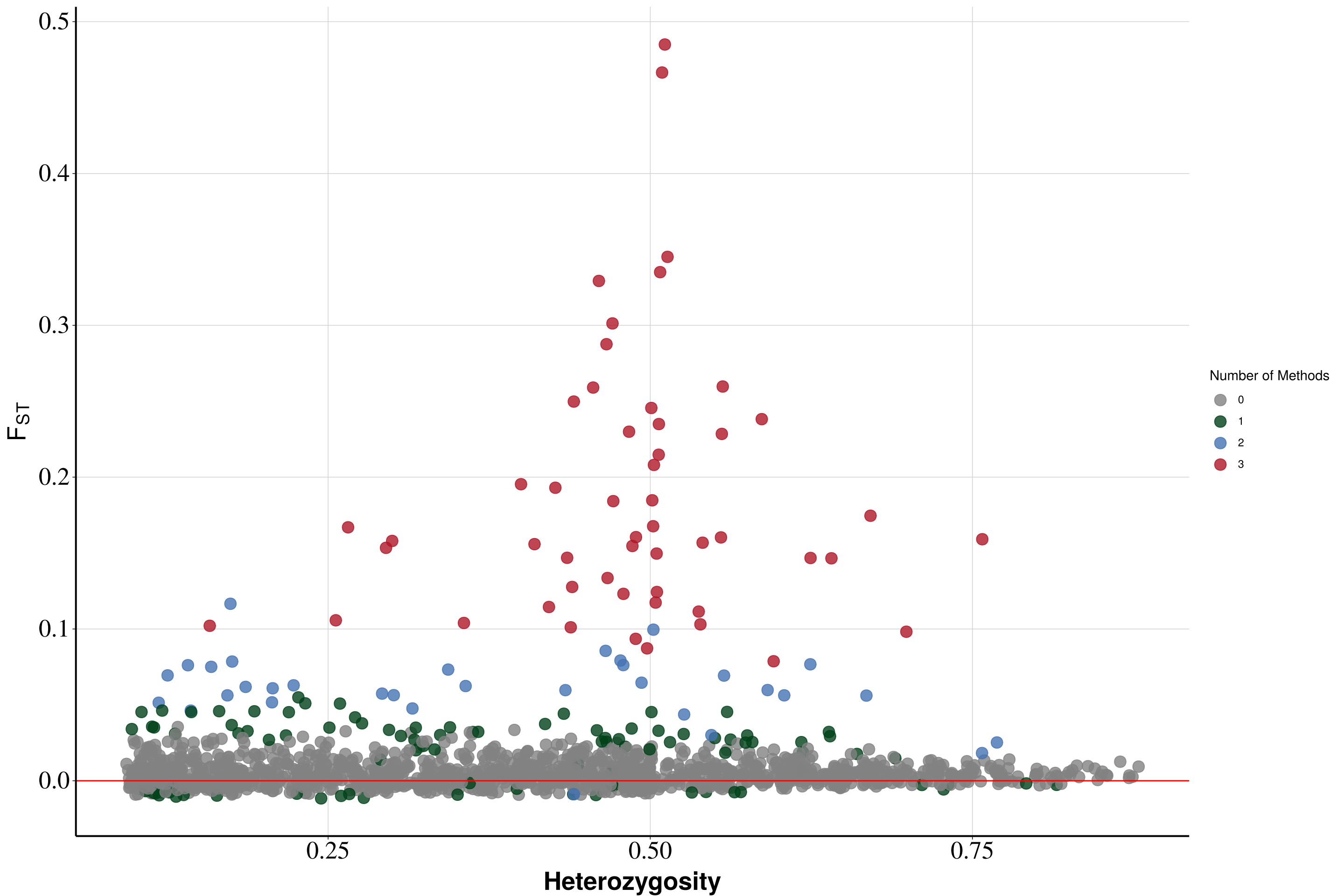

Supplement: Supplementary file 2 [file ECE3-9-3141-s002.pdf]
