# Supplementary material for: Evolution of population structure in an estuarine‐dependent marine fish
Source: Ecol Evol. 2019 Feb 26;9(6):3141–52. doi: 10.1002/ece3.4936 (PMC6434539; doi:10.1002/ece3.4936)
Supplement: Supplementary file 4 [file ECE3-9-3141-s004.docx]

**Supplemental Table 2.**  Estimates of pairwise *F_ST_* for 11 sampled localities, using datasets consisting of only neutral loci (*n* = 1,396) and only outlier loci (*n* = 143). Lower diagonal contains estimates of pairwise *F_ST_*; upper diagonal contains probability that *F_ST_* = 0. Significance was assessed by 10,000 permutations. Estimates in bold represent significant values following correction (FDR = 0.05).

| **Neutral Loci** | |  |  |  |  |  |  |  |  |  |  |
| --- | --- | --- | --- | --- | --- | --- | --- | --- | --- | --- | --- |
|  | LLM | MAT | SAB | MIS | APA | CEK | CHA | IND | HAR | WAS | SCA |
| LLM | -- | 0.69736 | 0.90536 | 0.85685 | 0.00059 | 0 | 0 | 0 | 0 | 0 | 0 |
| MAT | 0.00024 | -- | 0.9206 | 0.81982 | 0.0001 | 0 | 0 | 0 | 0 | 0 | 0 |
| SAB | -0.00032 | -0.0003 | -- | 0.51421 | 0.0002 | 0 | 0 | 0 | 0 | 0 | 0 |
| MIS | -0.00015 | -0.00004 | 0.00011 | -- | 0.09821 | 0 | 0 | 0 | 0 | 0 | 0 |
| APA | **0.00244** | **0.00242** | **0.00242** | 0.00114 | -- | 0.9207 | 0.60548 | 0 | 0 | 0 | 0 |
| CEK | **0.00364** | **0.00395** | **0.00402** | **0.00268** | -0.00021 | -- | 0.86684 | 0 | 0 | 0 | 0 |
| CHA | **0.00425** | **0.0033** | **0.0038** | **0.00302** | 0.00031 | -0.00005 | -- | 0 | 0 | 0 | 0 |
| IND | **0.00618** | **0.00603** | **0.00533** | **0.00577** | **0.00333** | **0.00509** | **0.00534** | -- | 0.01624 | 0.25077 | 0.12979 |
| HAR | **0.00509** | **0.0044** | **0.00532** | **0.00461** | **0.00448** | **0.0044** | **0.00456** | **0.00188** | -- | 0.09177 | 0.76418 |
| WAS | **0.00617** | **0.00567** | **0.00576** | **0.00427** | **0.0047** | **0.00477** | **0.00477** | 0.00083 | 0.00133 | -- | 0.85635 |
| SCA | **0.00556** | **0.00502** | **0.00512** | **0.00414** | **0.00344** | **0.00408** | **0.00416** | 0.00084 | 0.00013 | -0.00007 | -- |
| **Outlier Loci** | |  |  |  |  |  |  |  |  |  |  |
|  | LLM | MAT | SAB | MIS | APA | CEK | CHA | IND | HAR | WAS | SCA |
| LLM | -- | 0.04326 | 0.35096 | 0 | 0 | 0 | 0 | 0 | 0 | 0 | 0 |
| MAT | **0.00301** | -- | 0.03604 | 0 | 0 | 0 | 0 | 0 | 0 | 0 | 0 |
| SAB | 0.00098 | **0.00349** | -- | 0.19652 | 0 | 0 | 0 | 0 | 0 | 0 | 0 |
| MIS | **0.00843** | **0.00763** | 0.00152 | -- | 0 | 0 | 0 | 0 | 0 | 0 | 0 |
| APA | **0.13734** | **0.13072** | **0.12833** | **0.11353** | -- | 0 | 0 | 0 | 0 | 0 | 0 |
| CEK | **0.18713** | **0.18023** | **0.17803** | **0.1595** | **0.01426** | -- | 0.00584 | 0 | 0 | 0 | 0 |
| CHA | **0.21283** | **0.20997** | **0.20559** | **0.1863** | **0.02368** | **0.00462** | -- | 0 | 0 | 0 | 0 |
| IND | **0.09817** | **0.09027** | **0.09033** | **0.07656** | **0.03819** | **0.06033** | **0.07502** | -- | 0 | 0 | 0 |
| HAR | **0.08965** | **0.08254** | **0.08559** | **0.07448** | **0.05162** | **0.08165** | **0.09908** | **0.0125** | -- | 0.00109 | 0.00455 |
| WAS | **0.0889** | **0.08273** | **0.08457** | **0.07286** | **0.0447** | **0.07947** | **0.09241** | **0.0121** | **0.00784** | -- | 0.17167 |
| SCA | **0.08196** | **0.07367** | **0.07566** | **0.06409** | **0.04422** | **0.07269** | **0.08946** | **0.00677** | **0.00534** | 0.00183 | -- |
