# Supplementary material for: Evolution of population structure in an estuarine‐dependent marine fish
Source: Ecol Evol. 2019 Feb 26;9(6):3141–52. doi: 10.1002/ece3.4936 (PMC6434539; doi:10.1002/ece3.4936)
Supplement: Supplementary file 5 [file ECE3-9-3141-s005.docx]

**Supplemental Table 3**. Pairwise matrix of approximate coastline geographic distance. Distances were generated using Google Earth® and are reported in kilometers (km).

|  | **LLM** | **MAT** | **SAB** | **MIS** | **APA** | **CEK** | **CHA** | **IND** | **HAR** | **WAS** | **SCA** |
| --- | --- | --- | --- | --- | --- | --- | --- | --- | --- | --- | --- |
| **LLM** | -- |  |  |  |  |  |  |  |  |  |  |
| **MAT** | 290 | -- |  |  |  |  |  |  |  |  |  |
| **SAB** | 567 | 277 | -- |  |  |  |  |  |  |  |  |
| **MIS** | 1260 | 970 | 693 | -- |  |  |  |  |  |  |  |
| **APA** | 1687 | 1397 | 1120 | 427 | -- |  |  |  |  |  |  |
| **CEK** | 1961 | 1671 | 1394 | 701 | 274 | -- |  |  |  |  |  |
| **CHA** | 2248 | 1958 | 1681 | 988 | 561 | 287 | -- |  |  |  |  |
| **IND** | 2985 | 2695 | 2418 | 1725 | 1298 | 1024 | 737 | -- |  |  |  |
| **HAR** | 3306 | 3016 | 2739 | 2046 | 1619 | 1345 | 1058 | 321 | -- |  |  |
| **WAS** | 3391 | 3101 | 2824 | 2131 | 1704 | 1430 | 1143 | 406 | 85 | -- |  |
| **SCA** | 3537 | 3247 | 2970 | 2277 | 1850 | 1576 | 1289 | 552 | 231 | 146 | -- |
