# Supplementary material for: Evolution of population structure in an estuarine‐dependent marine fish
Source: Ecol Evol. 2019 Feb 26;9(6):3141–52. doi: 10.1002/ece3.4936 (PMC6434539; doi:10.1002/ece3.4936)
Supplement: Supplementary file 6 [file ECE3-9-3141-s006.docx]

**Supplemental Table 4.**  Environmental variables for each sampling locality, obtained from the National Estuarine Eutrophication Assessment database (<http://ian.umces.edu/neea/>). Descriptions of each variable can be found in metadata from the database.

| **Variable** | **LLM** | **MAT** | **SAB** | **MIS** | **APA** | **CEK** | **CHA** | **IND** | **HAR** | **WAS** | **SCA** |
| --- | --- | --- | --- | --- | --- | --- | --- | --- | --- | --- | --- |
| Estuary Area (km2) | 1308 | 1115 | 265 | 1581 | 593 | 165 | 502 | 866 | 39 | 88 | 85 |
| Tidal Fresh Zone Area (km2) | 0 | 2 | 8 | 0 | 46 | 7 | 1 | 0 | 5 | 11 | 1 |
| Mixing Zone Area (km2) | 27 | 918 | 257 | 1409 | 273 | 110 | 201 | 109 | 29 | 38 | 58 |
| Saltwater Zone Area (km2) | 1281 | 195 | 0 | 172 | 274 | 48 | 300 | 757 | 5 | 38 | 25 |
| Estuary Depth (m) | 0.76 | 1.41 | 2.49 | 2.43 | 1.81 | 1.17 | 1.63 | 0.77 | 1.96 | 3.35 | 4.99 |
| Estuary Perimeter (km) | 1413 | 790 | 355 | 553 | 402 | 345 | 567 | 1383 | 215 | 320 | 313 |
| Percent Estuary Open (%) | 0.04 | 0.76 | 0.17 | 1.99 | 2.99 | 11.89 | 2.64 | 0.21 | 1.63 | 1.75 | 0.83 |
| Catchment Area (km2) | 13165 | 121762 | 53674 | 4050 | 52214 | 25989 | 8134 | 3093 | 36962 | 12133 | 41116 |
| Catchment Mean Elev. (m) | 43 | 619 | 86 | 33 | 148 | 51 | 22 | 5 | 133 | 70 | 216 |
| Catchment Max Elev. (m) | 257 | 1374 | 236 | 124 | 1250 | 139 | 75 | 14 | 459 | 244 | 1679 |
| Catchment/Estuary Area Ratio | 10.1 | 109.2 | 202.5 | 2.6 | 88.1 | 157.5 | 16.2 | 3.6 | 947.7 | 137.9 | 483.7 |
| Total Land Cover (km2) | 13048.362 | 121234.778 | 51999.1897 | 39069.94 | 51483.7999 | 25837.6779 | 11447.736 | 2944.811 | 36754.50998 | 12058.9776 | 39857.31 |
| Population (#) | 616541 | 1432800 | 1230500 | 215299 | 2738086 | 417564 | 397072 | 471807 | 1681584 | 268166 | 3139518 |
| Pop / Estuary Area (#.km-2) | 471.4 | 1285 | 4643.4 | 136.2 | 4617.3 | 2530.7 | 791 | 544.8 | 43117.5 | 3047.3 | 36935.5 |
| Tide Height (m) | 0.4 | 0.2 | 0.47 | 0.51 | 0.58 | 0.76 | 0.65 | 0.32 | 1.9 | 1.93 | 1.45 |
| Tide Ratio | 0.53 | 0.14 | 0.19 | 0.21 | 0.32 | 0.65 | 0.4 | 0.42 | 0.97 | 0.58 | 0.29 |
| Stratification Ratio | 0.00146 | 0.04799 | 0.36975 | 0.00793 | 0.0976 | 0.10669 | 0.0085 | 0.00683 | 0.23917 | 0.02307 | 0.05799 |
| Percent Freshwater (%) | 0 | 0.2 | 3.1 | 0 | 7.8 | 4.1 | 0.2 | 0 | 12 | 12.6 | 1.4 |
| Percent Mixed Water (%) | 2.1 | 82.3 | 96.9 | 89.1 | 46.1 | 66.6 | 40.1 | 12.6 | 75.1 | 43.7 | 68.8 |
| Percent Seawater (%) | 97.9 | 17.5 | 0 | 10.9 | 46.2 | 29.3 | 59.7 | 87.4 | 13 | 43.7 | 29.8 |
| Average Salinity (psu) | 29 | 16 | 12 | 15 | 19 | 17 | 22 | 29 | 13 | 18 | 17 |
| Tidal Exchange (days) | 233 | 82 | 10 | 336 | 8 | 4 | 59 | 36 | 1 | 19 | 16 |
| Tidal Freshwater Flush (d) | 4 | 38 | 10 | 23 | 4 | 1 | 3 | 3 | 0 | 1 | 5 |
| Daily FW/Est Area (m.d-1) | 1.575 | 8.906 | 166.415 | 1.961 | 107.926 | 155.758 | 10.1 | 3.441 | 876.923 | 84.886 | 161.176 |
| Daily Freshwater (m3.d-1) | 2060000 | 9930000 | 44100000 | 3100000 | 64000000 | 25700000 | 5070000 | 2980000 | 34200000 | 7470000 | 13700000 |
| Flow / Estuary Area (m.d-1) | 1.575 | 8.906 | 166.415 | 1.961 | 107.926 | 155.758 | 10.1 | 3.441 | 876.923 | 84.886 | 161.176 |
| Total FW Volume (1.d-1) | 0.00074 | 0.00658 | 0.06743 | 0.00161 | 0.06044 | 0.13392 | 0.00655 | 0.00549 | 0.44801 | 0.02569 | 0.03256 |
| Daily Precipitation (m3.d-1) | 2.41E+06 | 3.27E+06 | 1.05E+06 | 6.88E+06 | 2.30E+06 | 571000 | 1.72E+06 | 3.06E+06 | 138000 | 306000 | 293000 |
| Daily Evaporation (m3.d-1) | 3.73E+06 | 2.86E+06 | 655000 | 3.80E+06 | 1.43E+06 | 417000 | 1.43E+06 | 2.38E+06 | 91800 | 203000 | 182000 |
| Daily Precip / Est Area (mm.d-1) | 1.843 | 2.933 | 3.962 | 4.352 | 3.879 | 3.461 | 3.426 | 3.533 | 3.538 | 3.477 | 3.447 |
| Daily Evap / Est Area (mm.d-1) | 2.852 | 2.565 | 2.472 | 2.404 | 2.411 | 2.527 | 2.849 | 2.748 | 2.354 | 2.307 | 2.141 |
| Flow (m3.d-1) | 2.06E+06 | 1.26E+07 | 5.97E+07 | 1.84E+06 | 6.56E+07 | 3.37E+07 | 2.46E+06 | 1.22E+06 | 3.88E+07 | 1.17E+07 | 3.60E+07 |
| Air Temp Mean (C) | 23.3 | 21.3 | 20.6 | 20.2 | 20.8 | 21.8 | 23.2 | 22.6 | 20.4 | 19.7 | 18.6 |
| Air Temp Std Dev (C) | 4.8 | 5.9 | 6.2 | 6.2 | 5.7 | 5 | 3.8 | 4 | 5.8 | 6.3 | 6.4 |
| Frost Days (#) | 3 | 9 | 15 | 21 | 16 | 13 | 1 | 3 | 28 | 29 | 41 |
| Wind Speed (m.sec-1) | 7.1 | 7 | 6.4 | 6.2 | 6 | 6 | 5.9 | 6.5 | 6.7 | 6.8 | 7.1 |
| Sea Surface Temp Mean (C) | 24.6 | 24.1 | 23.4 | 23.2 | 23.7 | 24 | 25.5 | 26.1 | 23.8 | 23.8 | 23.9 |
| Sea Surface Temp Std Dev (C) | 3.5 | 4.1 | 4.8 | 4.8 | 4.2 | 4 | 3.1 | 2.4 | 3.4 | 3.3 | 3.1 |
| Ocean Salinity Mean (psu) | 35.1 | 34.6 | 33.9 | 32.7 | 35.3 | 35.2 | 35.9 | 36.1 | 35.2 | 35.2 | 35.6 |
| Ocean Salinity Max (psu) | 36.6 | 36.2 | 35.8 | 35.1 | 36.4 | 36 | 36.3 | 36.3 | 35.8 | 35.8 | 36 |
| Ocean Salinity Min (psu) | 31.9 | 30.5 | 30.7 | 30.2 | 32.9 | 33.3 | 34.7 | 36 | 34.6 | 34.5 | 34.9 |
| Oceanic DIP (µM) | 0.2 | 0.21 | 0.32 | 0.27 | 0.13 | 0.12 | 0.1 | 0.16 | 0.17 | 0.16 | 0.16 |
| Oceanic NO3 (µM) | 0.12 | 0.14 | 0.7 | 1.95 | 0.87 | 0.59 | 0.58 | 0.27 | 0.29 | 0.29 | 0.33 |
| TSS (tonne.y-1) | 3.18E+06 | 1.14E+06 | 811000 | 563000 | 127000 | 175000 | 140000 | 39700 | 1960 | 17400 | 32200 |
| TN (kg.y-1) | 9.13E+06 | 9.13E+06 | 2.34E+07 | 1.62E+06 | 2.59E+07 | 5.78E+06 | 1.85E+06 | 1.61E+06 | 1.53E+07 | 6.40E+06 | 2.00E+06 |
| TP (kg.y-1) | 337900 | 830800 | 1.36E+06 | 166780 | 970300 | 2.46E+06 | 290780 | 187860 | 799800 | 440200 | 278380 |
| TSS/Est Area (tonne.km-2.y-1) | 2431.2 | 1022.4 | 3060.4 | 356.1 | 214.2 | 1060.6 | 278.9 | 45.8 | 50.3 | 197.7 | 378.8 |
| TN/Est Area (kg.km-2.y-1) | 6978.6 | 8186.5 | 88226.4 | 1027.2 | 43676.2 | 35042.4 | 3681.3 | 1859.1 | 391282.1 | 72704.5 | 23552.9 |
| TP/Est Area (kg.km-2.y-1) | 258.33 | 745.11 | 5135.47 | 105.49 | 1636.26 | 14917.58 | 579.24 | 216.93 | 20507.69 | 5002.27 | 3275.06 |
