# Supplementary material for: Evolution of population structure in an estuarine‐dependent marine fish
Source: Ecol Evol. 2019 Feb 26;9(6):3141–52. doi: 10.1002/ece3.4936 (PMC6434539; doi:10.1002/ece3.4936)
Supplement: Supplementary file 7 [file ECE3-9-3141-s007.docx]

**Supplemental Table 5.**  Summary of data filtering procedures: rows refer to each filtering step; columns refer to statistics for each step. For columns, ‘sites’ refers to individual polymorphisms (SNPs, indels, or complex polymorphisms), ‘loci’ refers to RAD contigs (each of which may contain multiple sites), and ‘Inds’ refers to individuals. ‘Start’, ‘End’, and ‘Removed’ refer, respectively, to the number of each unit before the filtering step, the number after the filtering step, and the number removed with the filter.

| **Filter** | **Start sites** | **End sites** | **Removed sites** | **Start loci** | **End loci** | **Removed loci** | **Start Inds** | **End Inds** | **Removed Inds** |
| --- | --- | --- | --- | --- | --- | --- | --- | --- | --- |
| Genotype depth < 10 | 430466 | 430466 | 0 | 33170 | 33170 | 0 | 568 | 568 | 0 |
| Mean site quality < 20 | 430466 | 361415 | 69051 | 33170 | 32988 | 182 | 568 | 568 | 0 |
| Mean site call rate < 0.5 | 361415 | 98309 | 263106 | 32988 | 8647 | 24341 | 568 | 568 | 0 |
| Ind depth < 10 & call rate < 0.25 | 98309 | 98309 | 0 | 8647 | 8647 | 0 | 568 | 551 | 17 |
| Mean site call rate < 0.75 | 98309 | 69484 | 28825 | 8647 | 5946 | 2701 | 551 | 551 | 0 |
| Ind call rate < 0.6 | 69484 | 69484 | 0 | 5946 | 5946 | 0 | 551 | 531 | 20 |
| Minor allele frequency < 0.05 | 69484 | 7890 | 61594 | 5946 | 3751 | 2195 | 531 | 531 | 0 |
| Discordant sites between duplicates | 7890 | 7811 | 79 | 3751 | 3740 | 11 | 531 | 531 | 0 |
| Remove duplicate individuals | 7811 | 7811 | 0 | 3740 | 3740 | 0 | 531 | 526 | 5 |
| Remove known hatchery individuals | 7811 | 7811 | 0 | 3740 | 3740 | 0 | 526 | 504 | 22 |
| Remove related individuals | 7811 | 7811 | 0 | 3740 | 3740 | 0 | 504 | 496 | 8 |
| dDocent_filters script | 7811 | 7351 | 460 | 3740 | 3633 | 107 | 496 | 496 | 0 |
| Decomposed to allelic primitives | 7351 | 7839 | -488 | 3633 | 3633 | 0 | 496 | 496 | 0 |
| Hardy-Weinberg equilibrium | 7839 | 7539 | 300 | 3633 | 3563 | 70 | 496 | 496 | 0 |
| Mean site call rate < 0.75 | 7539 | 7539 | 0 | 3563 | 3563 | 0 | 496 | 470 | 26 |
| Mean site call rate by locality < 0.85 | 7539 | 3689 | 3850 | 3563 | 1804 | 1759 | 470 | 470 | 0 |
| Mean site call rate overall < 0.95 | 3689 | 3642 | 47 | 1804 | 1784 | 20 | 470 | 470 | 0 |
| Haplotyping | 3642 | 2874 | 768 | 1784 | 1543 | 241 | 470 | 470 | 0 |
| Manual Inspection and Filtering | 2874 | 2860 | 14 | 1543 | 1539 | 4 | 470 | 462 | 8 |
