# Supplementary material for: Evolution of population structure in an estuarine‐dependent marine fish
Source: Ecol Evol. 2019 Feb 26;9(6):3141–52. doi: 10.1002/ece3.4936 (PMC6434539; doi:10.1002/ece3.4936)
Supplement: Supplementary file 11 [file ECE3-9-3141-s011.docx]

**Supplemental Table 9.** List of species with similar geographic patterns of genetic divergence. Atlantic/Gulf and NWG/NEG refers to species for which significant genetic divergence has been reported between the Atlantic and Gulf and the northwestern and northeastern Gulf, respectively.

| **Common name** | **Scientific Name** | **Family** | **Life History/Habitat Preference** | **Citation** |
| --- | --- | --- | --- | --- |
| **Atlantic/Gulf** |  |  |  |  |
| Red drum^a^ | *Sciaenops ocellatus* | Sciaenidae | Coastal demersal | This study |
| Black drum^b^ | *Pogonias cromis* | Sciaenidae | Coastal demersal | Leidig *et al.,* 2015 |
| Spotted seatrout^b,c^ | *Cynoscion nebulosus* | Sciaenidae | Coastal demersal | Seyoum *et al.,* 2018 |
| Sheepshead^b,c^ | [*Archosargus probatocephalus*](https://en.wikipedia.org/wiki/Archosargus_probatocephalus) | Sparidae | Coastal demersal | Seyoum *et al*., 2017 |
| Black sea bass^c^ | *Centropristis striata* | Serranidae | Inshore/offshore reef associated | Bowen and Avise, 1990 |
| Greater amberjack^c^ | *Seriola dumerili* | Carangidae | Coastal pelagic/reef associated | Gold and Richardson, 1998 |
| Gray snapper^b,c^ | *Lutjanus griseus* | Lutjanidae | Nearshore/reef associated | Gold *et al.,* 2009 |
| Southern flounder^b^ | *Paralichthys lethostigma* | Paralichthyidae | Coastal demersal | Anderson and Karel*,* 2012 |
| Blacknose shark^b,c^ | *Carcharhinus acronotus* | Carcharhinidae | Coastal demersal | Portnoy *et al.,* 2014 |
| Finetooth shark^b,c^ | *Carcharhinus isodon* | Carcharhinidae | Coastal demersal | Portnoy *et al.*2016 |
| Bonnethead shark^a,c^ | *Sphyrna tiburo* | Sphyrnidae | Coastal demersal | Portnoy *et al.,* 2015 |
| Dusky smooth-hound^b^ | *Mustelus canis* | Triakidae | Coastal demersal | Gold and Portnoy (2015) |
|  |  |  |  |  |
| **NWG/NEG** |  |  |  |  |
| Red drum^a^ | *Sciaenops ocellatus* | Sciaenidae | Coastal demersal | This study |
| Spotted seatrout^b,c^ | *Cynoscion nebulosus* | Sciaenidae | Coastal demersal | Seyoum *et al.,* 2018 |
| Sheepshead^b,c^ | [*Archosargus probatocephalus*](https://en.wikipedia.org/wiki/Archosargus_probatocephalus) | Sparidae | Coastal demersal | Seyoum *et al*., 2017 |
| Lane snapper^b,c^ | *Lutjanus synagris* | Lutjanidae | Nearshore/reef associated | Karlsson *et al.,* 2009 |
| Blacknose shark^b,c^ | *Carcharhinus acronotus* | Carcharhinidae | Coastal demersal | Portnoy *et al.,* 2014 |
| Finetooth shark^b,c^ | *Carcharhinus isodon* | Carcharhinidae | Coastal demersal | Portnoy *et al.* 2016 |
| Dusky smooth-hound^b^ | *Mustelus canis* | Triakidae | Coastal demersal | Gold and Portnoy (2015) |

^a^ Non-outlier and outlier SNPs (red drum); non-outlier SNPs (bonnethead shark)

^b^ Nuclear-encoded microsatellites

^c^ Mitochondrial DNA haplotypes or sequences

Citations

Anderson, J. D. and W. J. Karel (2012) Population genetics of southern flounder with implications for management. North American Journal of Fisheries Management 32: 656-662.

Bowen, B. W. and J. C. Avise (1990) Genetic structure of Atlantic and Gulf of Mexico populations of sea bass, menhaden, and sturgeon: Influence of zoogeographic factors and life-history patterns. Marine Biology 107: 371–381.

Gold JR, Richardson LR (1998) Population structure in greater amberjack, *Seriola dumerili*, from the Gulf of Mexico and the western Atlantic Ocean. Fishery Bulletin 96: 767-778.

Gold, J. R., E. Saillant, N. D. Ebelt, and S. Lem (2009) Conservation genetics of gray snapper (*Lutjanus griseus*) in U.S. waters of the northern Gulf of Mexico and western Atlantic Ocean. Copeia 2009: 277-286.

Gold, J. R. and D. S. Portnoy (2015) Stock structure of the smooth dogfish (*Mustelus canis*) in U.S waters. Final Report – Cooperative Research Program Grant No. NA12NMF4540083. Available from: NOAA Fisheries, Southeast Regional Office, http://sero.nmfs.noaa.gov/.

Karlsson S., E. Saillant, and J. R. Gold (2009) Population structure and genetic variation of lane snapper (*Lutjanus synagris*) in the northern Gulf of Mexico. Marine Biology 156: 1841-1855.

Leidig, J. M., V. R. Shervette, C. J. Mc Donough, and T. L. Darden (2015) Genetic population structure of black drum in U.S. waters. North American Journal of Fisheries Management 35:464-477.

Portnoy, D. S., C. M. Hollenbeck, D. M. Bethea, B. S., Frazier, J. Gelsleichter, and J. R. Gold (2016) Population structure, gene flow, and historical demography of a small coastal shark (*Carcharhinus isodon*) in U.S. waters of the Western Atlantic Ocean. ICES Journal of Marine Science 73: 2322-2332.

Portnoy, D.S., C. M. Hollenbeck, N. C. Belcher, W. B. Driggers III, B. S. Frasier, J. Gelsleichter, R> D. Grubbs, and J. R. Gold (2014) Contemporary population structure and post-glacial genetic demography in a migratory marine species, the blacknose shark, *Carcharhinus acronotus*. Molecular Ecology 23: 5480-5495.

Portnoy, D. S., J. B. Puritz, C. M. Hollenbeck, J. Gelsleighter, D. Chapman, and J. R. Gold (2015) Selection and sex-biased dispersal n a coastal shark: the influence of philopatry on adaptive variation. Molecular Ecology 24: 5877-5885.

Seyoum, S., R. S. McBride, C. Puchutulegui, J. Dutka-Gianelli, A. C. Alvarez, and K. Panzner (2017) Genetic population structure of sheepshead, *Archosargus probatocephalu*s (Sparidae), a coastal marine fish off the southeastern United States: multiple population clusters based on species-specific microsatellite markers. Bulletin of Marine Science 93: 691-713.

Seyoum, S., R. S. McBride, M. D. Tringali, V. L. Villanova, C. Puchutulegui, S. Gray, and N. V. Bibber (2018) Genetic population structure of the spotted seatrout (*Cynoscion nebulosus*): simultaneous examination of the mtDNA control region and microsatellite results. Bulletin of Marine Science 94: 47-71.
