# Supplementary material for: Evolution of population structure in an estuarine‐dependent marine fish
Source: Ecol Evol. 2019 Feb 26;9(6):3141–52. doi: 10.1002/ece3.4936 (PMC6434539; doi:10.1002/ece3.4936)
Supplement: Supplementary file 12 [file ECE3-9-3141-s012.docx]

**Supplemental Table 10.** Annual discharge rates of rivers and river complexes into the Gulf of Mexico. Placement of Mobile Bay in the NWG and rivers in Florida west of the Apalachicola River are for convenience as red drum in these rivers were not examined.

______________________________________________________________________________

*Northwestern Gulf* (west to east) *Northeastern Gulf* (east and south)

Bay/estuary Annual rate (m^3^/sec) Bay/estuary Annual rate (m^3^/sec)

*Texas*^1^ *Florida^4^*

Rio Grande 14.5 Perdido 21

Laguna Madre 29.1 Escambia 178

Nueces 23.0 Blackwater 10

Mission/Aransas 19.2 Yellow 33

Guadalupe 97.8 Choctawhatchee 204

Colorado/Lavaca 136.9 Apalachicola 470

East Matagorda 21.0 Ochlockonee 51

San Bernard 26.7 Aucilla 17

Brazos River 246.4 Suwanee 301

Trinity/San Jacinto^2^ 430.2 Withlacoochee 31

Sabine/Neches^2^ 547.6 Peace 33

Caloosahatchee 46

*Louisiana*^2^

Atchafalaya 1,642.4

Mississippi River 16,791.9

*Mississippi*^3^

All estuaries 884.0

*Alabama*^2^

Mobile Bay* 1,902.9

______________________________________________________________________________

^1^ Texas Water Development Board (2017) River Basins. Available at: <http://www.twdb.texas.gov/surfacewater/rivers/river_basins/index.asp>.

^2^ United States Geological Survey (1990) Largest rivers in the United States. Available at: https://pubs.usgs.gov/of/1987/ofr87-242/pdf/ofr87242.pdf.

^3^ Christmas, J. Y. and C. K. Eleuterius (1973) Phase II: Hydrology, pp. 74 -121 *In* J. Y. Christmas (ed.) Cooperative Gulf of Mexico Estuarine Inventory and Study, Mississippi Gulf Coast Research Laboratory, Ocean Springs, Mississippi, 434 pp.

^4^ McPherson, B. J. and B. J. Hammett (1991) Tidal rivers of Florida. Pages 31-46, in The Rivers of Florida (R. J. Livingston, ed.). Springer-Verlag, New York.
