# Supplementary material for: Evolution of population structure in an estuarine‐dependent marine fish
Source: Ecol Evol. 2019 Feb 26;9(6):3141–52. doi: 10.1002/ece3.4936 (PMC6434539; doi:10.1002/ece3.4936)
Supplement: Supplementary file 13 [file ECE3-9-3141-s013.docx]

# Supplementary Information

## ddRAD Library Preparation and Bioinformatics Filtering

Double digest RAD libraries were prepared as outlined in Portnoy et al. (2015) and sequenced on three lanes of an Illumina HiSeq 2000 DNA sequencer. Demultiplexing was performed with the program *process_radtags* from the Stacks package (Catchen et al. 2011), and read mapping and SNP calling were performed with the dDocent package (Puritz et al. 2014), following procedures outlined in (Hollenbeck et al. 2017). SNP loci were filtered using VCFtools (Danecek et al. 2011). Individual genotypes called with less than ten reads were excluded, as were all loci with a mean Phred score of <20. Next, loci with >50% missing data were excluded, followed by all individuals with a mean locus depth of less than ten and >75% missing data. Loci with >25% missing data were then excluded, followed by individuals with >40% missing data. Loci with a minor allele frequency of <0.05 were then excluded. Next, genotypes of five individuals, intentionally duplicated in different RAD libraries, were compared to look for genotype discordance consistent with systematic genotyping error. Loci which exhibited genotype differences across more than one set of replicates were discarded, and the individual from each set of duplicates that contained the most missing data across loci was removed from the data set. Following these steps, suspected first-generation, hatchery-derived individuals in the samples from Texas waters were removed from the dataset. Hatchery-derived individuals from the Texas stock-enhancement program were identified based on high genetic relatedness (*r* > 0.35; Yang et al. 2010) to at least one current hatchery brood fish; the latter had been genotyped with the same set of SNP loci for an ongoing study. Contigs were filtered further based on mean site depth, the ratio of quality to depth, strand representation, allelic balance in heterozygous individuals, and proper read pairing. A maximum depth cutoff of 300 reads per contig per individual was applied in the script. At this point, complex polymorphisms were decomposed to individual SNPs or indel loci, using the *vcfallelicprimitives* command in the vcflib package (https://github.com/vcflib/vcflib). Next, loci were tested for conformance to Hardy-Weinberg equilibrium within each locality; loci failing (*P* < 0.001) in more than two of the 13 samples were excluded. Individuals with >25% missing data were then excluded, followed by loci with >15% missing data in any single locality and more than five percent missing data across the entire data set. The dataset was then phased into constituent haplotypes using the program rad_haplotyper.pl (Willis et al. 2017) creating a dataset of multi-allelic, SNP containing haplotypes (loci). The program also removed any remaining indel-containing loci, loci with more than five individuals with too few or too many haplotypes given the SNP genotypes, and loci with < 95% successfully haplotyped individuals. The resulting Genepop file contained one diploid genotype per individual for each remaining locus. The necessity of sequencing individuals across multiple RAD libraries and HiSeq lanes can result in problematic loci that differ in allele frequencies due to systematic bias in the library preparation and sequencing process rather than due to population level processes (library effects, Meirmans 2015). To assess for library effects, the global *F_ST_* of each locus was plotted as a function of expected heterozygosity, using the program Lositan (Antao et al. 2008); loci that appeared as outliers in the distribution were inspected manually. As a second assessment, principal components analysis (PCA), implemented in the package adegenet (Jombart and Ahmed 2011) in R, was used to visualize the data, with individuals coded by RAD library rather than by sample locality. Loci exhibiting what possibly were library effects were then removed from the data set. Temporal stability of allele frequencies was assessed by comparing the two pairs of samples obtained from the same localities in different years (LLM 2008 vs. LLM 2014 and MAT 2008 vs. MAT 2015). Pairwise *F_ST_* was calculated in Arlequin (Excoffier and Lischer 2010) and significance assessed by a permutation test, with 10,000 permutations. Pairwise *F_ST_* did not differ significantly between years at either locality: LLM (*F_ST_* = -0.00017, *P* = 0.767) and MAT (*F_ST_* = 0.00048, *P* = 0.179). Samples were visualized with PCA, as above. Inspection of the PCA also confirmed the similarity of the temporal samples. The 2008 samples from LLM and MAT were then removed for the remainder of the analyses. In addition to establishing temporal stability, the PCA also revealed three distinct clusters of sample localities, corresponding to samples from the northwestern Gulf (LLM, MAT, SAB, and MIS), the northeastern Gulf (APA, CEK, and CHA), and the Atlantic (IND, HAR, WAS, and SCA). These regional groupings (northwestern Gulf - NWG, northeastern Gulf - NEG, and Atlantic - ATL) were used in subsequent hierarchical analyses of population structure and outlier detection.

## Outlier Detection

The data were screened for the presence of loci putatively under selection, using three *F_ST_* outlier-detection methods. The first approach used the fdist method (Beaumont and Nichols 1996), as implemented in Lositan, and employs coalescent simulations under a neutral island model to identify loci with *F_ST_* values that are either higher or lower than expected, given the observed heterozygosity. Lositan was run with 100,000 simulations and a false-discovery rate (FDR; Benjamin and Hochberg 1995) of 0.05. The second approach employed a modified version of the fdist method, implemented in Arlequin, which accounts for hierarchical population structure. Localities were placed into regional groupings, as suggested by the preliminary PCA, and the analysis was run with 50,000 simulations, with an FDR of 0.05. The last approach employed the program Bayescan (Foll and Gaggiotti 2008), which uses a Bayesian approach to estimate the posterior probability that each locus is under selection by comparing models that either incorporate or exclude the effects of selection. Bayescan was run with prior odds of selection relative to drift of 100:1 and a FDR of 0.05. Because loci with low minor allele frequencies can bias results of genome scans (Roesti et al. 2012), loci with a global, major-allele frequency above 0.95 were excluded from all three outlier-detection approaches. The dataset was then split into ‘neutral’ and ‘outlier’ components. The final outlier dataset consisted of all loci that had been identified as outliers under directional selection by at least one of the three approaches; the neutral dataset consisted of all remaining loci. All loci detected as outliers due to balancing selection had negative *F_ST_* values, suggesting that overall mean *F_ST_* in the dataset was too low to reliably detect balancing selection (Beaumont and Balding 2004; Narum and Hess 2011). For this reason, balancing selection ‘outliers’ were considered as neutral loci.

## Redundancy Analysis

Redundancy analysis was applied to investigate the influence of geographic distance and environmental variables on patterns of observed genetic variation. Redundancy analysis (RDA) combines correspondence analysis (CA) and multiple regression to measure the influence of a matrix of potential explanatory variables on a matrix of independent variables and can be used in a population-genetics context (Orsini et al. 2013; Vangestel et al. 2012). Geographic distance between sample localities was coded as a one-dimensional vector of approximate, linear coastline distance. Environmental data for each locality was obtained from the National Estuarine Eutrophication Assessment database (http://ian.umces.edu/neea/). In three cases (WAS, HAR, and CEK), data were not available for a bay or estuary sampled; data from the nearest bay or estuary (< 20 km away in all cases) were used as surrogates (Ossabaw Sound for WAS, Altamaha River for HAR, and Suwannee River for CEK). A set of 49 environmental variables was downloaded and standardized, to avoid bias caused by unequal variances. Standardization involved centering each variable value by subtracting the among-locality mean from each value, and scaling the centered variables by dividing each by the standard deviation of values among localities. Analysis was performed in R, based on a modified version (available at https://github.com/chollenbeck) of the method presented in Meirmans (2015). Genetic data were transformed into a set of synthetic variables describing the among-locality component of genetic variation, using PCoA as implemented in the package adegenet. The ten largest principal coordinate axes were retained and used as dependent variables in the RDA model. To avoid overfitting the model due to correlation of independent variables, forward selection of geographic and environmental variables was conducted with the *ordistep* function in the vegan package. This function iteratively adds and drops variables from the model and assesses significance of a change by a permutation test to select variables that best explain the data. This was applied to the geographic and environmental variables separately, using the full dataset. Following forward selection of variables, RDA analysis was conducted with the *rda* function (vegan). The *varpart* function (vegan) was used to partition the total genetic variance into components explained by geography, environment, and a ‘shared’ component, which represents variance explained by both geography and environment that cannot be decomposed into one or the other. Significance of the overall model and of each variance component was conducted with the *anova.cca* function (vegan), using 1,000 permutations. While variance components attributable to independent variables alone (geography or environment, in this case) are testable under an RDA framework, the shared component of variance is not because it can only be estimated from the other components and thus has zero degrees of freedom (Borcard et al. 2011). The RDA analysis was conducted separately with datasets containing only neutral loci and only outlier loci.

## Detection of Genomic Regions under Selection

The most recent version of the red drum genetic linkage map (Hollenbeck et al. 2017) was developed with the same ddRAD methodology, meaning that a subset of the RAD contigs genotyped in this study were segregating in an informative fashion in the mapping crosses, and were thus able to be assigned a genomic position. To further investigate genomic patterns of divergence, global *F*_ST_ (with individuals grouped by region) as well as regional, pairwise *F*_ST_ (NWG-NEG, NWG-ATL, NEG-ATL) was calculated, using Weir and Cockerham’s (1984) estimator, as implemented in the pegas package (Paradis 2010) in R. *F*_ST_ values for loci that had been incorporated successfully into the linkage map were then plotted against genomic position. A set of high confidence outliers was identified by selecting loci that met at least one of the following criteria: i) the locus was identified by all three outlier-detection approaches, ii) the locus was within two cM of another outlier, and iii) the locus had a global *F_ST_* of at least 0.1.

## Identification of Candidate Genes

To develop a list of candidate genes potentially under the influence of selection, the RAD reference sequences for the high-confidence outlier loci were compared to the annotated draft genome of the large yellow croaker (*Larimichthys crocea*), the closest relative to red drum for which a genome sequence was available (Wu et al. 2014). The assembly of the large yellow croaker genome (GenBank assembly L_crocea_1.0) was downloaded, along with information regarding the genomic positions of annotated proteins. The croaker genome was converted to a BLAST database, using the *makeblastdb* function in NCBI’s Standalone BLAST package (Camacho et al. 2009). The blastn algorithm was applied to align high-confidence outlier loci to the croaker genome. For each RAD locus, the longest hit over 100 bp and with the lowest e-value was retained. For each *L. crocea* scaffold containing a BLAST hit from an outlier RAD locus, the annotated gene closest to the RAD locus with the highest *F_ST_* was recorded. To facilitate further analysis of gene functions, each candidate gene was manually annotated with the gene symbol of its human orthologue, using the GeneCards database (http://www.genecards.org/). Gene network analysis and gene-ontology (GO) term-enrichment analysis for biological process were then performed with the String database (http://www.string-db.org). Significance of GO term enrichment analysis was assessed at an FDR of 0.05. Because the actual target of selection may not be the gene closest to an *F_ST_* outlier, the list of potential candidate genes was expanded by recording every annotated gene in the *L. crocea* genome where at least part of the gene boundary was within 100kb of each outlier RAD locus. The web service AmiGO (http://amigo.geneontology.org/) was used to extract all human genes associated with significant GO terms in the previous analysis, and all candidate genes were queried against this list.

## References

Antao TA, Lopes A, Lopes RJ, Albano BP, Luikart G. 2008 Lositan: A workbench to detect molecular adaptation based on a Fst-outlier method. *BMC Bioinformatics* **9**. doi:[10.1186/1471-2105-9-323](https://doi.org/10.1186/1471-2105-9-323).

Beaumont MA, Nichols RA. 1996 Evaluating loci for use in the genetic analysis of population structure. *Proc. R. Soc. B* **263**, 1619–1626.

Beaumont MA, Balding DJ. 2004 Identifying adaptive genetic divergence among populations from genome scans. *Mol. Ecol.* **13**, 969–980.

Benjamini Y, Hochberg Y. 1995 Controlling the false discovery rate: a practical and powerful approach to multiple testing. *J. R. Stat. Soc.* **57**, 289–300.

Borcard D, Gillet F, Legendre P. 2011 *Numerical Ecology with R*. doi:[10.1017/CBO9781107415324.004](https://doi.org/10.1017/CBO9781107415324.004).

Camacho C, Coulouris G, Avagyan V, Ma N, Papadopoulos J, Bealer K, Madden TL. 2009 BLAST+: Architecture and applications. *BMC Bioinformatics* **10**, 1–9.

Catchen JM, Amores A, Hohenlohe P, Cresko W, Postlethwait JH. 2011 Stacks: building and genotyping loci de novo from short-read sequences. *G3 Genes|Genomes|Genetics* **1**, 171–182.

Danecek P, Auton A, Abecasis G, [Albers CA](https://www.ncbi.nlm.nih.gov/pubmed/?term=Albers%20CA%5BAuthor%5D&cauthor=true&cauthor_uid=21653522), [Banks E](https://www.ncbi.nlm.nih.gov/pubmed/?term=Banks%20E%5BAuthor%5D&cauthor=true&cauthor_uid=21653522), [DePristo MA](https://www.ncbi.nlm.nih.gov/pubmed/?term=DePristo%20MA%5BAuthor%5D&cauthor=true&cauthor_uid=21653522), [Handsaker RE](https://www.ncbi.nlm.nih.gov/pubmed/?term=Handsaker%20RE%5BAuthor%5D&cauthor=true&cauthor_uid=21653522), [Lunter G](https://www.ncbi.nlm.nih.gov/pubmed/?term=Lunter%20G%5BAuthor%5D&cauthor=true&cauthor_uid=21653522), [Marth GT](https://www.ncbi.nlm.nih.gov/pubmed/?term=Marth%20GT%5BAuthor%5D&cauthor=true&cauthor_uid=21653522), [Sherry ST](https://www.ncbi.nlm.nih.gov/pubmed/?term=Sherry%20ST%5BAuthor%5D&cauthor=true&cauthor_uid=21653522), [McVean G](https://www.ncbi.nlm.nih.gov/pubmed/?term=McVean%20G%5BAuthor%5D&cauthor=true&cauthor_uid=21653522), [Durbin R](https://www.ncbi.nlm.nih.gov/pubmed/?term=Durbin%20R%5BAuthor%5D&cauthor=true&cauthor_uid=21653522). 2011 The variant call format and VCFtools. *Bioinformatics* **27**, 2156–2158.

Excoffier L, Lischer H. 2010 Arlequin suite ver 3.5: a new series of programs to perform population genetics analyses under Linux and Windows. *Mol. Ecol. Res.* **10**, 564–567.Foll M, Gaggiotti O. 2008 A genome-scan method to identify selected loci appropriate for both dominant and codominant markers: A Bayesian perspective. *Genetics* **180**, 977–993.

Hollenbeck CM, Portnoy DS, Wetzel D, Sherwood TA, Samollow PB, Gold JR. 2017 Linkage mapping and comparative genomics of red drum (*Sciaenops ocellatus*) using next-generation sequencing. *G3 – Genes Genomes Genetics* **7**, 843-850.

Jombart T, Ahmed I. 2011 adegenet 1.3-1: New tools for the analysis of genome-wide SNP data. *Bioinformatics* **27**, 3070–3071.

Meirmans PG. 2015 Seven common mistakes in population genetics and how to avoid them. *Mol. Ecol.* **24**, 3223–3231.

Narum SR, Hess JE. 2011 Comparison of F_ST_ outlier tests for SNP loci under selection. *Mol. Ecol. Res.* **11** Suppl 1, 184–94.

Orsini L, Mergeay J, Vanoverbeke J, De Meester L. 2013 The role of selection in driving landscape genomic structure of the waterflea *Daphnia magna*. *Mol. Ecol.* **22,** 583–601.

Paradis E. 2010 Pegas: An R package for population genetics with an integrated-modular approach. *Bioinformatics* **26**, 419–420.

Portnoy DS, Puritz JB, Hollenbeck CM, Gelsleichter J, Chapman D, Gold JR. 2015 Selection and sex-biased dispersal in a coastal shark: the influence of philopatry on adaptive variation. *Mol. Ecol.* **24**, 5877–5885.

Puritz JB, Hollenbeck CM, Gold JR. 2014 dDocent: a RADseq, variant-calling pipeline designed for population genomics of non-model organisms. *PeerJ* **2**, e431.

Roesti M, Salzburger W, Berner D. 2012 Uninformative polymorphisms bias genome scans for signatures of selection. *BMC Evol. Biol.* **12**, 94.

Vangestel C, Mergeay J, Dawson DA, Callens T, Vandomme V, Lens L. 2012 Genetic diversity and population structure in contemporary house sparrow populations along an urbanization gradient. *Heredity* **109**, 163–172.

Weir BS, Cockerham CC. 1984 Estimating F-statistics for the analysis of population structure. *Evolution* **38**, 1358–1370.

Willis SC, Hollenbeck CM, Puritz JB, Gold JR, Portnoy DS. 2017 Haplotyping RAD loci: an efficient method to filter paralogs and account for physical linkage. *Mol. Ecol. Res.* **17**, 955-965 doi:10.1111/1755-0998.12647.

Wu C, Zhang D, Kan M, Lv Z, Zhu A, Su Y, Zhou D, [Zhang](https://www.semanticscholar.org/author/Jianshe-Zhang/2710014) J, [Zhang](https://www.semanticscholar.org/author/Zhou-Zhang/39431863) Z, [Xu](https://www.semanticscholar.org/author/Meiying-Xu/40553288) M, [Jiang](https://www.semanticscholar.org/author/Lihua-Jiang/2219252) L, [Guo](https://www.semanticscholar.org/author/Baoying-Guo/39269782) B, [Wang](https://www.semanticscholar.org/author/Ting-Wang/39945663) T, [Chi](https://www.semanticscholar.org/author/Changfeng-Chi/37981822) C, [Mao](https://www.semanticscholar.org/author/Yong-Mao/40026649) Y, [Zhou](https://www.semanticscholar.org/author/Jiajian-Zhou/6661988) J, [Yu](https://www.semanticscholar.org/author/Xinxiu-Yu/5879659) X, [Wang](https://www.semanticscholar.org/author/Hailing-Wang/2549581) H, [Weng](https://www.semanticscholar.org/author/Xiaoling-Weng/5545520) X, [Jin](https://www.semanticscholar.org/author/Jason-Gang-Jin/7703286) JG, [Ye](https://www.semanticscholar.org/author/Junyi-Ye/6468843) J, [He](https://www.semanticscholar.org/author/Lin-He/40218563) L, [Liu](https://www.semanticscholar.org/author/Yun-H.-Liu/39367758) YH. 2014 The draft genome of the large yellow croaker reveals well-developed innate immunity. *Nat. Com.* **5**, 5227. doi:[10.1038/ncomms6227](https://doi.org/10.1038/ncomms6227).

Yang J., Benyamin B, Mcevoy BP, Gordon S, Henders AK, Dale R, Madden PA, [Heath AC](https://www.ncbi.nlm.nih.gov/pubmed/?term=Heath%20AC%5BAuthor%5D&cauthor=true&cauthor_uid=20562875), [Martin NG](https://www.ncbi.nlm.nih.gov/pubmed/?term=Martin%20NG%5BAuthor%5D&cauthor=true&cauthor_uid=20562875), [Montgomery GW](https://www.ncbi.nlm.nih.gov/pubmed/?term=Montgomery%20GW%5BAuthor%5D&cauthor=true&cauthor_uid=20562875), [Goddard ME](https://www.ncbi.nlm.nih.gov/pubmed/?term=Goddard%20ME%5BAuthor%5D&cauthor=true&cauthor_uid=20562875), [Visscher PM](https://www.ncbi.nlm.nih.gov/pubmed/?term=Visscher%20PM%5BAuthor%5D&cauthor=true&cauthor_uid=20562875). 2010. Common SNPs explain a large proportion of heritability for human height. *Nat, Genet* **42,** 565–69. doi:[10.1038/ng.608](https://doi.org/10.1038/ng.608).
