# Supplementary material for: Evolution of population structure in an estuarine‐dependent marine fish
Source: Ecol Evol. 2019 Feb 26;9(6):3141–52. doi: 10.1002/ece3.4936 (PMC6434539; doi:10.1002/ece3.4936)
Supplement: Supplementary file 14 [file ECE3-9-3141-s014.docx]

Captions for Supplemental Tables and Figures

Supplemental Table Captions

**Supplemental Table 1**: Sampling data for individuals (localities) used in the study.

**Supplemental Table 2**: Estimates of pairwise *F_ST_* for 11 sampled localities, using datasets consisting of only neutral loci (*n* = 1,396) and only outlier loci (*n* = 143). Lower diagonal contains estimates of pairwise *F_ST_*; upper diagonal contains probability that *F_ST_* = 0. Significance was assessed by 10,000 permutations. Estimates in bold represent significant values following correction (FDR = 0.05).

**Supplemental Table 3**: Pairwise matrix of approximate coastline geographic distance. Distances were generated using Google Earth® and are reported in kilometers (km).

**Supplemental Table 4**: Environmental variables for each sampling locality, obtained from the National Estuarine Eutrophication Assessment database (<http://ian.umces.edu/neea/>). Descriptions of each variable were extracted from metadata included in the database.

**Supplemental Table 5**: Summary of data filtering procedures: rows refer to each filtering step; columns refer to statistics for each step. For columns, ‘sites’ refers to individual polymorphisms (SNPs, indels, or complex polymorphisms); ‘loci’ refer to RAD contigs (each of which may contain multiple sites); and ‘Inds’ refers to individuals. ‘Start’, ‘End’, and ‘Removed’ refer, respectively, to the number of each unit before the filtering step, the number after the filtering step, and the number removed with the filter.

**Supplemental Table 6:** Map location of 45 high-confidence outliers: LG – linkage group, Pos – map position (in cM), Cluster – outlier cluster number.

**Supplemental Table 7**: Summary of significant Gene Ontology (GO) terms. FDR is the false discovery rate for the test. Proteins are the list of protein coding genes in each significant GO category that were located within 100kb of an outlier locus.

**Supplemental Table 8**: Summary of all candidate genes within 100 kb of an outlier locus.

**Supplemental Table 9**: List of species with similar geographic patterns of genetic divergence. Atlantic/Gulf and NWG/NEG refers to species for which significant genetic divergence has been reported between the Atlantic and Gulf and the northwestern and northeastern Gulf, respectively.

**Supplemental Table 10**: Annual discharge rates of rivers and river complexes into the Gulf of Mexico. Placement of Mobile Bay in the NWG and rivers in Florida west of the Apalachicola River are for convenience as red drum in these rivers were not examined.

Supplemental Figure Captions

**Supplemental Figure 1**: Principal components analysis (PCA), using all loci (*n* = 1,539), and including two pairs of temporal samples from Lower Laguna Madre (LLM) and West Matagorda Bay (MAT).

**Supplemental Figure 2**: A plot of *F_ST_* (y-axis) and expected heterozygosity (x-axis) for each locus in the dataset. Each point represents a locus and the color of each point indicates the number of outlier-detection methods that determined a locus to be an outlier.
